# Kinetics of Nickel Diffusion into Austenitic Stainless Steels AISI 304 and 316L and Calculation of Diffusion Coefficients

**DOI:** 10.3390/ma16206783

**Published:** 2023-10-20

**Authors:** Šárka Bukovská, Jaromír Moravec, Martin Švec

**Affiliations:** Department of Engineering Technology, Faculty of Mechanical Engineering, Technical University of Liberec, 46117 Liberec, Czech Republic; jaromir.moravec@tul.cz (J.M.); martin.svec@tul.cz (M.Š.)

**Keywords:** diffusion coefficient, nickel, stainless steel, AISI 304, AISI 316L, diffusion bonding

## Abstract

Diffusion bonding has many advantages, but it also has its specifics. When creating heterogeneous joints, problems arise with the creation of intermetallic phases. For this reason, an interlayer is needed to prevent the creation of these unfavorable phases. It is important to ensure that the interlayer is of sufficient thickness to prevent the elements from diffusing through the entire interlayer and the intermetallic phases from being formed again. Conversely, too thick an interlayer causes an increase in the heterogeneity of the bond properties. The creation of the initial diffusion bonds in a heterogeneous diffusion joint of AISI 304 and AISI 316L steel with a 0.2 mm thick nickel interlayer was made in a Gleeble 3500. The experiments to determine the diffusion kinetics were carried out in a vacuum furnace, with subsequent evaluation by EDX (Energy Dispersive X-ray Spectroscopy) analysis. Subsequently, the diffusion coefficients of nickel into both steels were determined, and generalized equations were formulated to calculate the diffusion coefficients for temperatures in the range of 950 to 1150 °C and holding times in the range of 3600 to 18,000 s. Equations are also given to determine the width of the diffused zone between each steel and the Ni interlayer.

## 1. Introduction

Diffusion bonding is a method of joining homogeneous and heterogeneous solid-state joints that has some advantages over fusion welding methods. These include, for example, a significant decrease in the burning of alloying elements, a very small heat-affected zone, but also joining materials that are not weldable by fusion methods due to differences in thermal conductivity and coefficient of linear expansion [1], or joining metals and non-metals [2].

However, the creation of heterogeneous joints increases the risk of the formation of intermetallic phases, which are brittle and decrease the strength of the joints [3]. The formation of intermetallic phases can be eliminated by using interlayers [4]. The joining of titanium and stainless steel has been studied, for example, by scientists from India, and their results were published in [5]. They dealt with the diffusion bonding of commercially pure titanium and AISI 304 stainless steel with a nickel interlayer. The joining of commercially pure titanium and AISI 304 steel was also discussed in the paper [6]. Furthermore, the joining of Ti and stainless steel AISI 304 was dealt with in the article [7], where the authors focused on the influence of diffusion bonding time on the microstructure and mechanical properties of the joints. The study of interfacial reactions and strength properties of diffusion joints between a titanium alloy and a micro-duplex steel is described in [8], where the authors used nickel as an interlayer.

In another research study [9], the authors focus on diffusion bonding with a 0.1 mm thick Ni interlayer between martensitic steel (410 grade) and copper. Copper jaws were used to join the materials; welding temperatures were 800–950 °C, pressure 12 Mpa, and time 60 min, and vacuum 1.33 × 10^−8^ MPa. Another work [10] also dealt with welding with a nickel interlayer, which was used to weld Ti alloy (Ti6Al4V) and stainless steel AISI 301.

The welding of Ti and AISI 304 stainless steel with a nickel interlayer was also studied in [11], where the authors joined these two materials using the friction welding method. They reported that no intermetallic phases were formed when Ti and 304 steel were joined using a Ni interlayer. Another study [12] compares joints formed by diffusion bonding and friction welding. The joint was formed by a Ti alloy (Ti6Al4V) and AISI 304 stainless steel with an interlayer of copper. Friction-welded joints had better joint strength (up to 520 MPa compared to 282 MPa for diffusion joints), and processing time was also minimal (36 min) compared to diffusion joints (127 min).

As mentioned above, other metals can also be used as an interlayer or a combination of different metals (multilayer) to create diffusion joints. In the work [13], the authors describe the joining of titanium to austenitic stainless steel using an Nb/Cu/Ni multilayer. The work [14] deals with the influence of Cu, Ni, and Ag interlayers on the microstructure and mechanical properties of titanium and stainless steel joints.

From the research, it is evident that the use of nickel as an interlayer in heterogeneous joints is common; however, in these articles [5,6,8,15], the authors mention that at higher temperatures or longer holding times, elements such as Ti, Cr, and Fe were diffused through Ni or other interlayer to form intermetallic phases. Therefore, it is useful to know how fast the individual elements will diffuse into each other at the specific process parameters—temperature and bonding time. Although there are many studies dealing with the creation of heterogeneous joints, the choice of the interlayer thickness is not discussed in them, and the investigations carried out could not find any studies dealing with this problem.

This is the reason why this paper deals with the determination of Ni diffusion coefficients in AISI 304 and AISI 316L austenitic stainless steels. For heterogeneous joints that contain a nickel interlayer, the calculation of diffusion coefficients can be complicated. However, some studies have focused on this problem and provided information about diffusion coefficients for various heterogeneous joints with a nickel interlayer. For example, in the work mentioned above [15], the authors deal with the joining of titanium and AISI 304 stainless steel by using a nickel interlayer with a thickness of 300 μm. The joints were created at temperatures of 800–950 °C with a uniaxial pressure of 3 MPa under vacuum. The authors present the intrinsic diffusion coefficients for titanium: D_Ti_ = 5.5 × 10^−14^ m^2^·s^−1^ at 900 °C and D_Ti_ = 9 × 10^−14^ m^2^·s^−1^ at 800 °C. The intrinsic diffusion coefficients for iron α DFe-α = 5 × 10^−15^ m^2^·s^−1^ at 900 °C and for nickel D_Ni_ = 3 × 10^−17^ m^2^·s^−1^ at 800 °C were taken from other studies that dealt with diffusion coefficients [16,17,18].

The research presented in this paper is focused on the determination of nickel diffusion kinetics and the calculation of diffusion coefficients. Research on diffusion bonding with the nickel interlayer has been described in the following works [15,19,20]. Nickel diffusion was investigated in frequently used materials in diffusion bonding, namely AISI 304 and AISI 316L steel. Diffusion joints with 304 steel have been described in [21,22,23,24] and with 316L steel, for example, in [25]. The calculation of the diffusion coefficients was based on Fick’s laws, and the diffusion kinetics were determined by EDX analysis. The knowledge of the diffusion coefficients for specific applications is important not only to determine the optimal interlayer thickness but also to optimize the welding time or to determine the critical concentrations at which intermetallic phases are formed according to the binary diagrams.

Determining the optimum interlayer thickness is very important for diffusion joints. If the thickness is insufficient, the elements will diffuse, and unwanted intermetallic phases will form. On the other hand, if the interlayer is too thick, heterogeneity and, therefore, diversity in the mechanical and physical properties of the joint will increase significantly. All this has a consequent effect on the limitation of the application use of the joints, both in terms of the environment and conditions in which they work, as well as in terms of the type and especially the magnitude of the working loads.

The aim of this article is to show the diffusion of Ni into the austenitic stainless steels AISI 304 and AISI 316L and to determine the diffusion coefficients for the temperatures of 950, 1050, and 1150 °C. These are values that have not yet been published anywhere.

In addition, the paper is structured in such a way that researchers can use it to experimentally determine diffusion coefficients for any combinations of heterogeneous joints.

## 2. Materials and Methods

Two austenitic stainless steels were used to determine the Ni diffusion coefficient. The first material was AISI 304, 1.4301 according to ISO 10027-2 [26], and X5CrNi18-10 according to ISO 10027-1 [27]. This is an austenitic stainless steel that has good corrosion resistance in a common environment. The samples were made from round-rolled rods with a diameter of 12 mm. The chemical composition was measured using a Bruker Q4 Tasmann spectrometer (Karlsruhe, Germany). The chemical composition shown in Table 1 is calculated from the average of 7 measurements. Furthermore, the chemical composition in Table 1 is defined by EN10088-1 [28].

The second material was austenitic stainless steel AISI 316L, 1.4404 according to ISO 10027-2 [26], and X2CrNiMo17-3-2 according to ISO 10027-1 [27]. The samples were again made from a 12 mm diameter round-rolled rod. Table 2 shows the chemical composition of the steel and the chemical composition as defined by EN10088-1 [28]. The chemical composition was also measured at 7 places using a Bruker Q4 Tasmann spectrometer (Karlsruhe, Germany), and the average value of these measurements is shown in Table 2.

The mechanical properties of the delivered materials YS (yield strength), UTS (ultimate tensile strength), A_g_, and A_40_ (the tensile strength where the original length of the test sample was 40 mm) were measured at room temperature (RT). A TIRA Test 2300 (TIRA GmbH, Schalkau, Germany) was used for testing. The static tensile test was realized according to EN ISO 6892-1 [29] with a loading rate of 1 mm/min up to achieving yield strength and then 15 mm/min. The mechanical properties were determined for both base materials.

**Figure 1:**
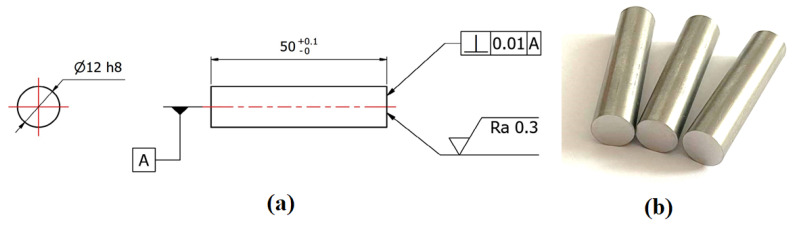
Drawing of the sample used for diffusion bonding in the Gleeble 3500 (**a**), example of real samples for diffusion bonding (**b**).

The samples of both steels were cylinders of 50 mm in length and 12 mm in diameter. Figure 1 shows the drawing of the samples (a) and the real samples made of AISI 304 and AISI 316L steel (b). Diffusion bonding of the samples was realized in a thermal–mechanical simulator, Gleeble 3500 (Dynamic System Inc., New York, NY, USA), where the samples were bonded at a temperature of 1050 °C, 13 MPa pressure, and 15 min. It was a heterogeneous joint with a nickel interlayer that was 0.2 mm thick. The purpose of the process was to create only initial diffusion bonds between the two materials and the Ni interlayer so that the sample would not fracture during further processing. In addition, the surface roughness was measured for both the contact surfaces of the AISI 304 and AISI 316L steel, as well as for the nickel foil. Roughness affects the quality and strength of the joint. Surface irregularities will not allow ideal contact in the contact area. The components then only touch at specific points. These irregularities can be eliminated by decreasing the roughness and, furthermore, by applying a clamping force during joining. The roughness on the contact surfaces of the samples was measured with a MITUTOYO SV-2000N2 SURFTEST contact profilometer (Mitutoyo, Kanagawa, Japan), and the roughness of the nickel foil was measured on a KEYENCE VK-X1100 3D confocal microscope (Keyence, Itasca, IL, USA). Control of the device and evaluation of the results were realized by the MultiFileAnalyser VK-H1XMD 2019 software (Keyence, Itasca, IL, USA).

The samples had to be carefully prepared for diffusion bonding. Both steels were supplied in the form of a 12 mm diameter cold-rolled bar. From this bar, 50 mm long samples were cut, and the faces of the samples were ground to achieve the specified surface roughness and surface parallelism (see Figure 1a).

**Figure 2:**
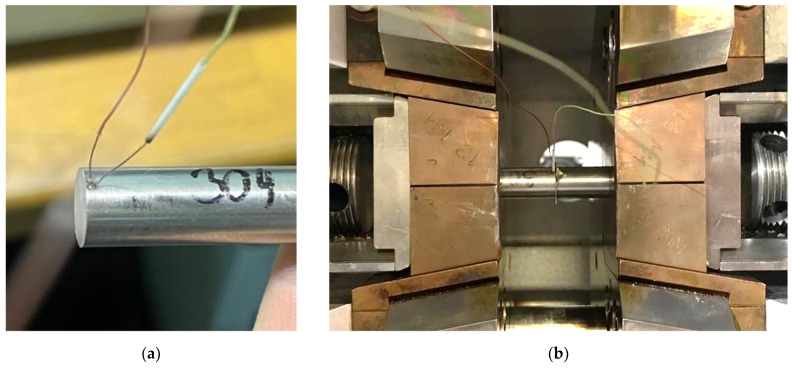
Example of the thermocouple welded to the sample boundary and (**a**) the sample with the nickel interlayers clamped in the chamber of the Gleeble device (**b**).

A K-type thermocouple was welded to one sample. Because the maximum bonding temperature was 1050 °C, these K-type thermocouples can be used without any problems. The welded thermocouple can be seen in Figure 2a. After the thermocouple was welded, the samples made of AISI 304 steel on one side and AISI 316L steel on the other side were put into full-contact copper jaws and placed in the chamber of the Gleeble device (Dynamic System Inc., New York, NY, USA), as shown in Figure 2b. Both the sample surfaces and the nickel foil surfaces were degreased and cleaned, and then two layers of foil were pressed between the two samples. Two foils were used to achieve a final interlayer thickness of 0.2 mm.

**Figure 3:**
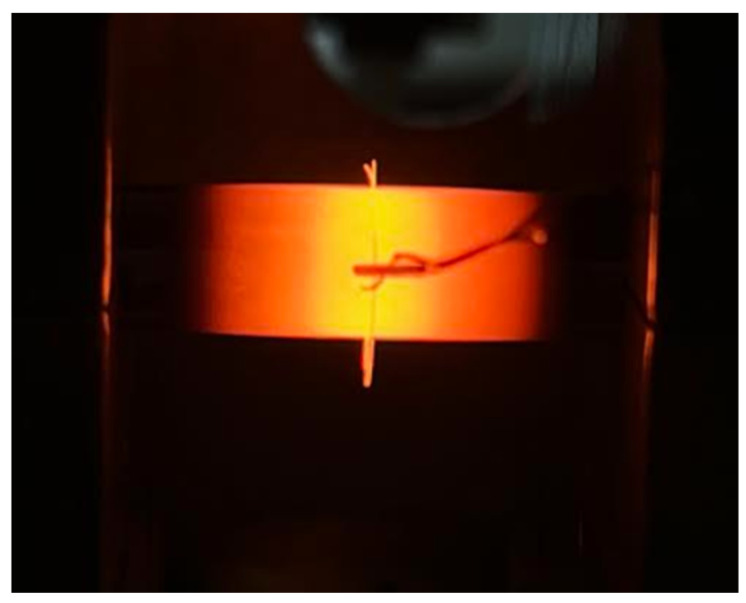
Sample during diffusion bonding—temperature 1050 °C.

After clamping the samples, the chamber was vacuumed, and a programmed cycle could be run to connect the samples and the nickel foils. The welding parameters were: temperature 1050 °C, holding time at this temperature 15 min, heating and cooling rate to this temperature 5 °C·s^−1,^ and pressure 13 MPa. Figure 3 shows a sample heated to 1050 °C.

The experiment evaluating the diffusion of Ni into both stainless steels was realized in a Reetz vacuum furnace (HTM Reetz GmbH, Berlin, Germany). In the vacuum furnace, temperature treatments with maximum temperatures of 950 °C, 1050 °C, and 1150 °C were applied. The heating rate was graded and the same for all experiments (0.8 °C·min^−1^ to 80 °C; 1.4 °C·min^−1^ to 160 °C; 1.8 °C·min^−1^ to 220 °C; 3 °C·min^−1^ to 600 °C, and 7 °C·min^−1^ in the temperature interval 600 to 1150 °C). The holding time at temperature was 1 h and 5 h. The cooling rate of the samples was also constant for all experiments at 5 °C·min^−1^. During the heat treatment, the vacuum in the furnace was 7 × 10^−5^ mbar. No more pressure was applied to the samples with initial diffusion bonds.

Subsequent evaluation of diffusion kinetics was realized on a Tescan Mira 3 scanning electron microscope (SEM) (Tescan Orsay Holding a.s., Brno, Czech Republic). EDX (Energy Dispersive X-ray Spectroscopy, Tescan Orsay Holding a.s., Brno, Czech Republic) analysis was used to obtain the concentration of individual elements as a function of distance for each type of heat treatment. The measurements were realized with an accelerating voltage of 10 kV.

## 3. Experiment and Results

### 3.1. Determination of Mechanical Properties

The mechanical properties of the material were measured by static tensile testing, and the results are shown in Table 3. The mechanical properties of the material were measured for both AISI 304 and AISI 316L steel.

### 3.2. Diffusion Bonding in Gleeble 3500

On the contact surfaces of the AISI 304 test samples, a roughness of Ra = 0.259 µm was measured, and for the AISI 316L samples, Ra = 0.260 µm. These values are the average of 5 measurements taken. For nickel foil, the roughness Sa = 0.101 µm was measured.

**Figure 4:**
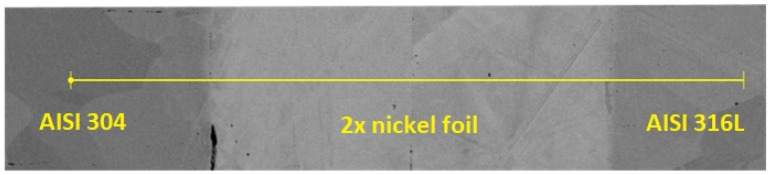

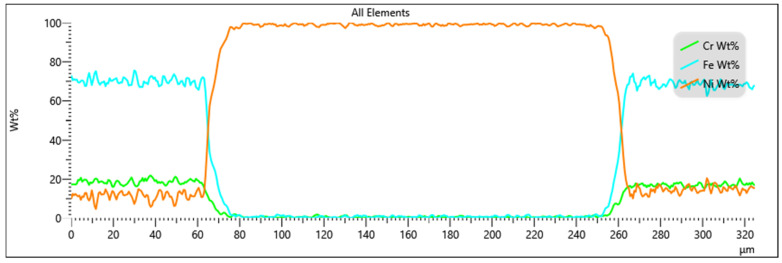
Heterogeneous joint with nickel interlayer in initial state (after bonding in Gleeble 3500).

Diffusion bonding was only used to create initial diffusion bonds so that the sample would not fracture during subsequent cutting and diffusion could take place across the initial bonds. This sample was cut on a metallographic machine and prepared for evaluation under an electron microscope. Using this evaluation, the primary diffusion bonds were detected, and EDX analysis confirmed that there was very little diffusion into the base material. For AISI 304 steel, it was only 1 µm, and for AISI 316L steel, it was 1.5 µm. Even with a perfectly sharp interface, a width of at least 0.5 µm would be measured, due to the SEM/EDX resolution being around 1 µm. More detailed results of the EDX analysis of the sample after diffusion bonding in the Gleeble 3500 are shown in Figure 4.

### 3.3. Evaluation of Diffusion Kinetics

The bonded and cut samples were then put into a vacuum furnace, where diffusion proceeded under defined boundary conditions. Three temperatures were chosen for testing: 950 °C, 1050 °C, and 1150 °C, and two holding times: 1 h and 5 h. Each sample was individually put into the vacuum furnace and was therefore processed under the conditions shown in Table 4.

After sample heat treatment, the concentration of elements (Cr, Fe, and Ni) was determined by EDX analysis. The effect of temperature and time on the diffusion process can be evaluated from the nickel concentration curves. Seven linear EDX analyses were performed on each sample to exclude sample inhomogeneity. The results of all EDX analyses were then used to determine the diffusion coefficient.

**Figure 5:**
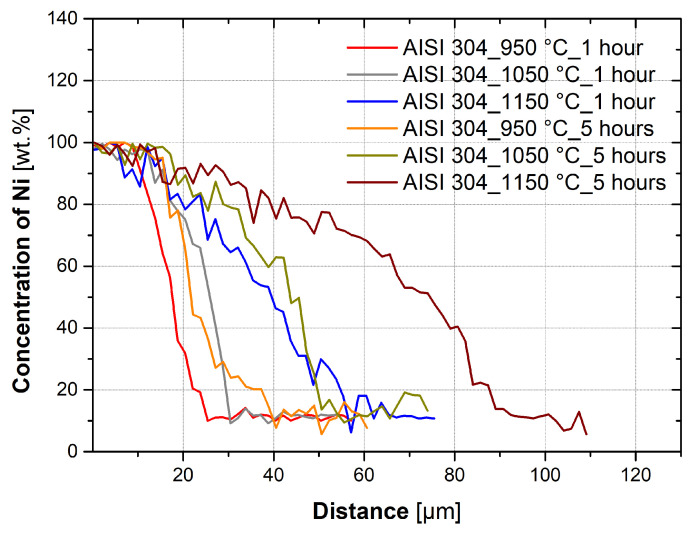
Comparison of AISI 304 samples with heat treatment with different temperatures, holding time of 1 h, and holding time of 5 h.

**Figure 6:**
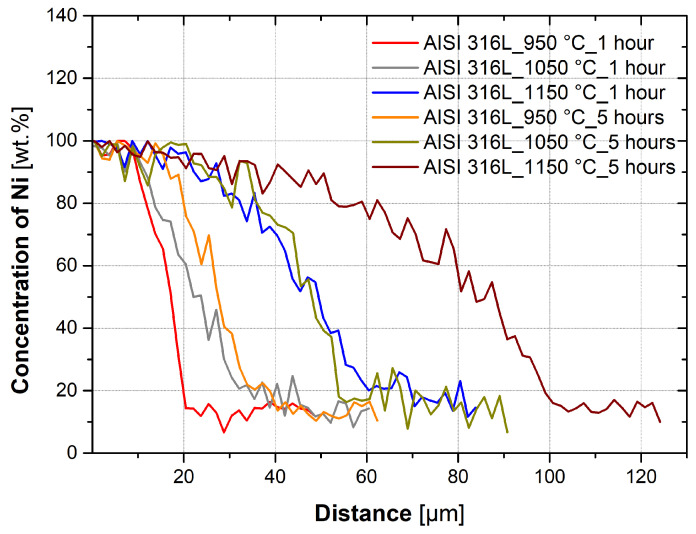
Comparison of AISI 316L samples with heat treatment with different temperatures, holding time of 1 h, and holding time of 5 h.

Figure 5 shows the effect of temperature on nickel diffusion for AISI 304 steel with a holding time of 1 h and a holding time of 5 h. Figure 6 shows the effect of temperature on AISI 316L steel for a holding time of 1 h and a holding time of 5 h. The data shown in the graphs are from only one linear analysis. From the graphs, it is evident that the higher temperature of treatment has caused the diffusion of nickel to a larger depth of the base material. It is evident from the graphs that the higher temperature and longer holding time of the heat treatment resulted in the diffusion of nickel to a greater depth of the base material than was the case for the 1 h holding time.

**Figure 7:**
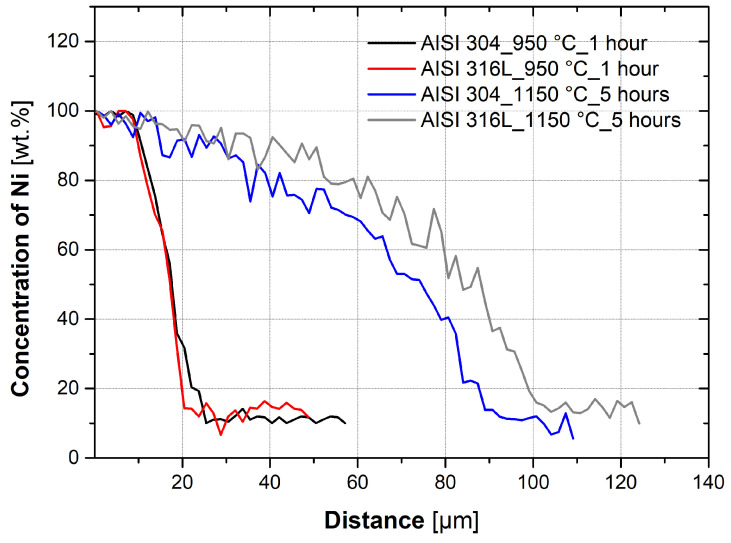
Comparison of concentration gradients between AISI 304 and AISI 316L steel.

Furthermore, a comparison of two extreme heat treatments (950 °C_1 h and 1150 °C_5 h) for both materials (AISI 304 and AISI 316L) was performed. This comparison is shown in the graphs in Figure 7. It is evident from the graph that the difference in heat treatments is significant; the depth to which the nickel diffused is significantly greater at higher temperatures and for a longer time. The graph also shows that nickel diffuses into both steels similarly; the difference in the nickel concentration gradient is minimal.

Using induction or resistance heating, high heating rates can be achieved at the holding temperature at which diffusion joints are formed. This can minimize the effect of heating on the total width of the diffusion zone. However, in the industrial production of diffusion joints, special equipment with indirect heating of the sample is generally used. In these cases, the heating and cooling rates are in the order of units of °C·min^−1^. Therefore, a vacuum furnace was chosen for the experiments to achieve realistic heating and cooling conditions. However, diffusion already occurs during heating and cooling. Separate experiments were realized to study the effect of heating and cooling in the vacuum furnace on the total width of the diffusion zone. These were composed only of heating to the set holding temperature, zero holding time at that temperature, and subsequent cooling. Identical rates were used for heating and cooling, as described in Section 2.

### 3.4. Calculation of Diffusion Coefficients

**Figure 8:**
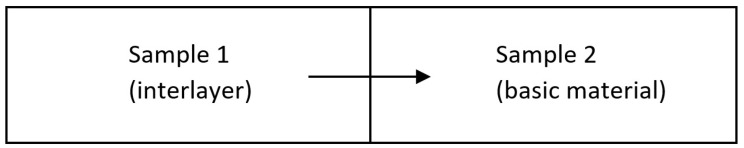
Diffusion across the interfacial interface.

The diffusion coefficients for the specified temperatures can be calculated from the previous data. This is the case of diffusion across the planar interfacial interface of a diffusion joint formed by joining two materials (Figure 8), in which the initial concentrations of the diffusing element are *c*_1_ and *c*_2_. The concentration of the diffusing element at distance *x* from the interface at time *t* is *c*. This configuration can be used to determine the diffusion coefficient D for a specific temperature from known values of the concentrations *c*_1_ and *c*_2_ and from the measured concentration *c* for a distance *x* and time *t.* This is diffusion in an unlimited space, i.e., in terms of diffusion in the interval (−∞ < 0 < ∞).

The initial condition for this case: before and at the start of diffusion (*t* = 0). The concentration at each place (*x* < 0) is *c* = *c*_1_. At each place (*x* > 0), the concentration is *c* = *c*_2_. Thus:

*c* = *c*_1_, *x* < 0, *t* = 0 (1)

*c* = *c*_2_, *x* > 0, *t* = 0 (2)

Boundary condition in this case: at the planar interface (*x* = 0), a constant concentration ½(*c*_1_ + *c*_2_) is maintained at all times (*t* > 0) Thus:

½(*c*_1_ + *c*_2_), *x* = 0, *t* > 0 (3)

**Figure 9:**
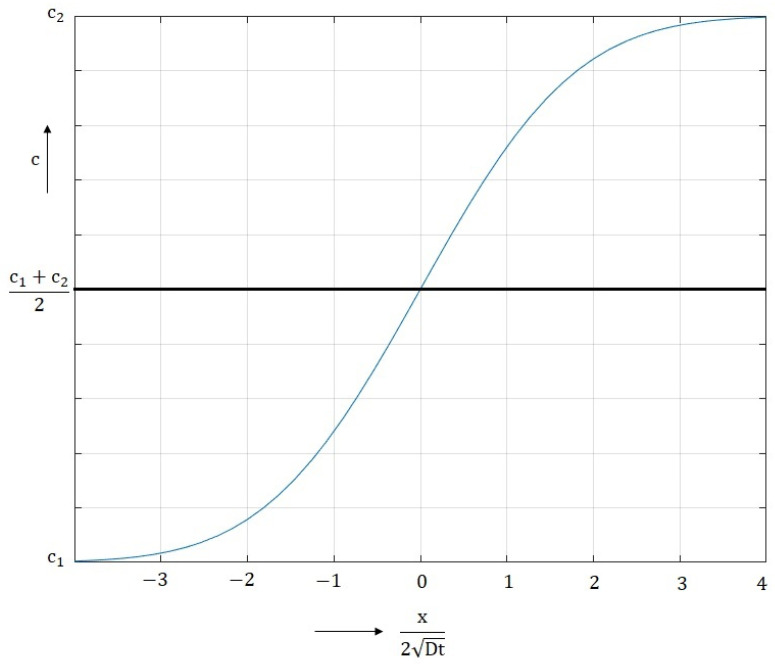
Graphical representation of Equations (4) and (5).

The concentration *c* at distance *x* and time *t* is determined from Equations (4) and (5), which are based on Fick’s 2nd law. Figure 9 is a graphical representation of them.

(4) $$c=c_{1}+\frac{c_{2}-c_{1}}{2}\left[1-erf(z)\right]\;\mathrm{for}\;c<\frac{c_{1}+c_{2}}{2}$$

(5) $$c=c_{1}+\frac{c_{2}-c_{1}}{2}\left[1+erf(z)\right]\;\mathrm{for}\;c>\frac{c_{1}+c_{2}}{2}$$

If sample 1 is an interlayer that diffuses into the base material (sample 2), then this is a case of diffusion of the joint with the interlayer, so the specific mathematical solution for calculating the diffusion coefficient D is described below.

From Equations (4) and (5), the form of Equation (6) can be written, which describes the entire shape of the curve shown in Figure 9.

(6) $$c\left(x,t\right)=\frac{c_{2}+c_{1}}{2}-\frac{c_{2}-c_{1}}{2}*erf\left(\frac{x-x_{0}}{2\sqrt{Dt}}\right)$$

where *c* is the concentration at location *x* and time *t*, *c*_1_ is the concentration in the base material, *c*_2_ is the concentration in the interlayer, *D* is the diffusion coefficient, *t* is time, *x* is the distance from the interface, and *x*_0_ is the distance at the interface (*x*_0_ = 0).

By modifying Equation (6), the form of Equation (7) can be obtained:

(7) $$ierf\;\left(\frac{c\left(x,t\right)-\frac{c_{2}+c_{1}}{2}}{\frac{c_{2}-c_{1}}{2}}\right)=\frac{x-x_{0}}{2\sqrt{Dt}}$$

where *ierf* (*z*) is the inverse of the error function. The left side of the equation can be renamed—see Equation (8).

(8) $$y=ierf\;\left(\frac{c\left(x,t\right)-\frac{c_{2}+c_{1}}{2}}{\frac{c_{2}-c_{1}}{2}}\right)$$

and farther:

(9) $$k=\frac{1}{2\sqrt{Dt}}$$

These modifications produce the equation of line (10), which should follow the experimentally obtained curve as closely as possible.

(10) y=k∗x+q

where *y* is the y coordinate of the point, *k* is the directive of the line, *x* is the x coordinate of the point, and *q* is the displacement of the line.

From Equation (10), the directive of the line can be found, and the value of *k* can be substituted into Equation (9). The diffusion coefficient can then be determined according to Equation (11).

(11) $$D=\frac{1}{{4k}^{2}t}$$

From these equations, the diffusion coefficients were calculated and are presented in Table 5. The table shows the median of the 7 calculated *D* from the 7 measured line data for each heat treatment.

After calculating the diffusion coefficients, a statistical analysis, a two-factor ANOVA with repetition, was used to determine if temperature and time had a statistically significant effect on diffusion. For a more accurate result, the statistical analysis was performed on the 7 calculated D, not the median, which is shown in Table 5. The significance level of the test was β = 5%, and the results of the two-factor ANOVA analysis of variance are shown in Table 6. The statistical evaluation confirmed that temperature and time have a statistically significant effect.

**Figure 10:**
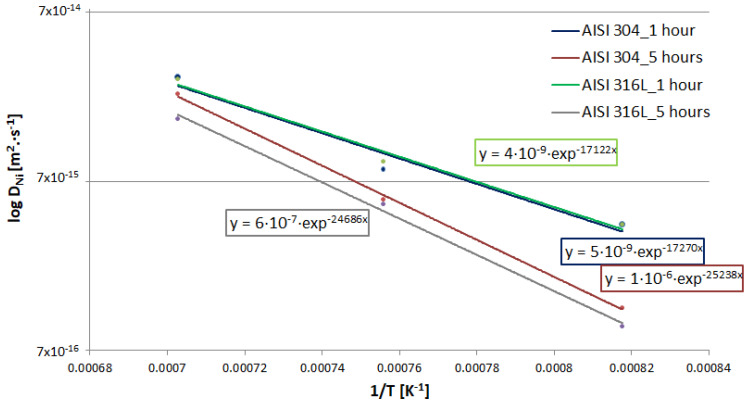
Dependence of log D_Ni_ on 1/T for AISI 304 and 316L steels and both 1 h and 5 h.

From the basic laws of diffusion processes, it is clear that *D* is very dependent on temperature. As the temperature increases, the diffusion rate increases. This temperature dependence can be represented by Equation (12).

(12) $$D=D_{0}\cdot {exp}^{\frac{-Q_{m}}{R_{m}T}}$$

where *D* is the diffusion coefficient, *D*_0_ is a partial factor depending on the state of the crystal lattice and the frequency of diffusing atoms, *Q_m_* is the activation energy, *R_m_* is the mole gas constant, and *T* is the absolute temperature. Equation (12) shows the exponential dependence of *D* on −1/*T*. Figure 10 shows the dependence of log D_Ni_ on 1/T with the corresponding equations.

**Figure 11:**
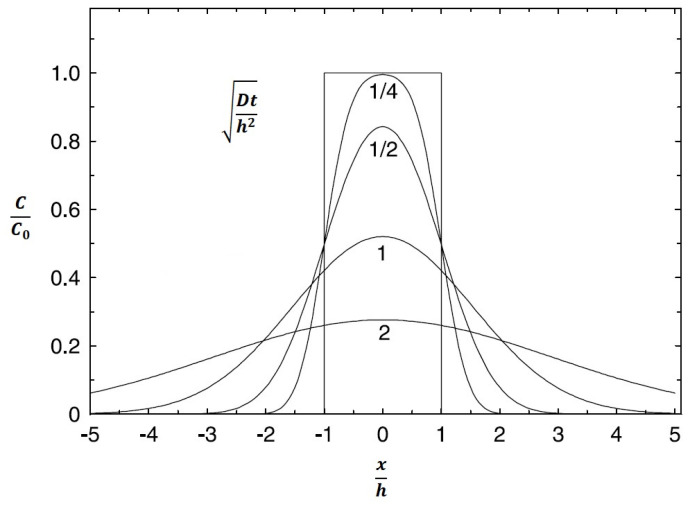
Diffusion from a 2h wide sheet for different values $\frac{\sqrt{Dt}}{h}$.

In the experiments described in this study, an interlayer of sufficient thickness was used (always 100% Ni in the middle of the joint), and therefore the joint can be imaginary divided for calculation and the diffusion coefficients for each part can be calculated separately. If the diffusion coefficient is already known, another case of diffusion calculation can be applied to represent the real case of a diffusion joint. Such a case is illustrated in Figure 11.

In this arrangement, a plate (interlayer) of width 2 h, having the same initial concentration, is connected to two half-spaces, which in the real case are two bars of pure material. The diffusion field in this case can be expressed by Equation (13).

(13) $$c\left(x,t\right)=\frac{c_{0}}{2}\left[erf\left(\frac{x+h}{2\sqrt{Dt}}\right)+erf\left(\frac{x-h}{2\sqrt{Dt}}\right)\right]$$

where *c* is the concentration at location *x* and time *t*, *c*_0_ is the concentration in the interlayer (for *x* = 0), *x* is the distance from the center of the interlayer, *h* is half the thickness of the interlayer, D is the diffusion coefficient, and *t* is time. For *x* = 0, Equation (14) can be used.

(14) $$c\left(t\right)=c_{0}*erf\left(\frac{h}{2\sqrt{Dt}}\right)=c_{0}*erf\left(\frac{1}{2\sqrt{\frac{Dt}{h^{2}}}}\right)$$

For curve 1/4 of Figure 11, when the nickel concentration in the interlayer is *c*_0_ = 100%, Equation (15) can be used.

(15) $$\sqrt{\frac{Dt}{h^{2}}}=\frac{1}{4}\;then:\;\frac{1}{2\sqrt{\frac{Dt}{h^{2}}}}=\frac{1}{2\frac{1}{4}}=2\to erf\left(2\right)=1\to c\left(t\right)=c_{0}=100\;\%$$

Equation (15) can be used to check the relevance of the determined diffusion coefficient D_Ni_ and determine with what validity the calculated D_Ni_ can be used for further calculations. Equation (15) can be used to calculate the depth of diffusion under specific conditions (temperature and time), and the results can be compared with the real values obtained by EDX analysis. The experimentally determined diffusion depths to assess the effects of heating and cooling and each heat treatment and both materials are given in Table 7. The results of the calculated diffusion depths according to Equation (15) are shown in Table 8. Table 9 then shows the absolute values of the difference between the real and calculated depths in micrometers.

## 4. Discussion

The work aimed to study the diffusion kinetics of nickel into austenitic stainless steels (AISI 304 and AISI 316L) and the subsequent determination of the diffusion coefficients of nickel into individual steels for a defined temperature and time range.

From the experiments and calculations, it is possible to form a few conclusions. The kinetics of diffusion, and thus the depth of diffusion of nickel into the austenitic steel, are significantly affected by the temperature at which diffusion takes place, as can be seen in Figure 5 and Figure 6. The effect of temperature on diffusion bonding is described by the authors in their works [5,15], where they show that at higher temperatures, intermetallic phases are formed because the diffusion of the individual elements is faster and thus the concentration at which the intermetallic phases are formed. For both AISI 304 and AISI 316L steels, nickel diffused to a depth 2–3 times larger than at 950 °C and 1.5–2 times larger than at 1050 °C.

The kinetics of the diffusion of nickel into the austenitic steel parts depend not only on the temperature but also on the holding time at that temperature. Figure 7 compares two limit cases of Ni diffusion at 950 °C and a holding time of 1 h and at 1150 °C and a holding time of 5 h. It is evident from the results that higher temperatures and longer periods have a significant effect on the diffusion depth of Ni. It is also evident that nickel diffuses into the two used austenitic steels, AISI 304 and AISI 316L, at approximately the same rate, although AISI 316L has approximately 5% more Ni and 2.2% more Mo. The effect of temperature and time on Ni diffusion was confirmed by the results of a two-factor analysis of variance (ANOVA) with repetition.

The intensity of diffusion that occurs during heating to the welding temperature and during cooling from this temperature is exponentially dependent on the value of the maximum temperature (950, 1050, and 1150 °C). In industrial diffusion bonding, indirect radiative heating is generally used with heating and cooling rates of the sample in the order of units of °C·min^−1^. In this case, for austenitic steels, the effect of heating to 950 °C and subsequent cooling from this temperature is 5.6–7.3% on the total diffusion width of Ni. At 1050 °C, it is then 6.8–12.6%, and at 1150 °C, it is even 11.7–23.8% of the total diffusion width of Ni. The result also shows a clear difference for the holding times of 1 and 5 h, as the total diffusion width is larger for the holding time of 5 h (see Table 7).

In the work [15], the authors present the diffusion coefficient for nickel, D_Ni_ = 3 × 10^−17^ m^2^·s^−1^. However, for practical application, this information is not sufficient because it is necessary to know the diffusion coefficients of the element into specific materials. Equation (12) shows that the diffusion coefficient is exponentially dependent on −1/T, and thus it is necessary to have data about diffusion coefficients, ideally in the form of temperature dependences. The obtained temperature dependences of the diffusion coefficients of Ni into austenitic steels are based on the Arrhenius equation. Therefore, it would probably be sufficient to give only Equations (16) and (17), from which *D*_0_ and *Q* can be obtained. The aim of this paper was to show a method of determining diffusion coefficients in a broader context so that other researchers can simply duplicate the above process and determine the diffusion coefficients at a specific temperature for any combinations of diffusion joints with interlayers, and also to determine the diffusion depths from which the interlayer thickness can be determined for heterogeneous joints (Equations (18) and (19)).

Based on the experiments (Figure 10), generalized Equations (16) and (17) were formulated to determine the value of the diffusion coefficients of Ni into individual steels with sufficient accuracy. Equation (16) can be used to determine the specific value of the diffusion coefficient of Ni into AISI 316L steel for the temperature range 1223.15–1423.15 K, where *T* is the temperature (K) at which diffusion takes place. At these studied temperatures (950–1150 °C), a fully austenitic microstructure is expected, and delta ferrite is not formed as predicted by equilibrium phase diagrams of steels. The diffusion coefficients can be extrapolated up to temperatures where delta ferrite starts to form.

(16) $$logD=3\times 10^{-7}\cdot exp(-20,904\cdot \frac{1}{T})$$

Equation (17) can be used to determine the specific value of the diffusion coefficient of Ni into AISI 304 steel, also in the temperature range 1223.15–1423.15 K.

(17) $$logD=5\times 10^{-7}\cdot exp(-21,254\cdot \frac{1}{T})$$

To verify the accuracy of the values of diffusion coefficients, the depths of diffusion of Ni into the tested austenitic steels were calculated and compared with the measured depths from the EDX analysis. The results are presented in Table 7 and Table 8. Table 9 shows the difference between the diffusion depths. Thus, the calculated diffusion depths were different from approximately 5% to 28.6%, with the largest differences occurring at 950 °C. At higher temperatures, the percentage differences were lower because the diffusion gradient was more stabilized and moderate due to the higher temperatures. Although the deviation from the real value seems to be quite big, realistically, the difference in diffusion area width at 950 °C is not more than 6.7 µm, and at 1150 °C it is not more than 11.6 µm. The higher deviations between the experimentally determined and calculated diffusion depths are due to the diffusion occurring during heating and cooling of the sample, which is not taken into account in the calculated diffusion depths. If the diffusion during heating and cooling is taken into account in the depth calculations, the deviations will be between 0.40 and 27.98%. The decrease occurred mainly at 950 °C, where the deviations were above 24% in all cases, but after taking into account the diffusion during heating and cooling, the deviations decreased to 18%; only for material 316L and a holding time of 5 h, the deviation was 23%.

What is important is knowledge of the specific diffusion zone width between the interlayer and the base material. This is determined from knowledge of the diffusion coefficient of Ni into the individual steels, as shown in Equation (15). For AISI 316L steel, it can generally be determined for temperatures (*T*) in the range 1223.15–1423.15 K and for times (*t*) in the range 1 to 5 h according to the formulated Equation (18), where *h* is the width of the diffusion zone (m); *T* is the temperature at which diffusion takes place (K); and *t* is the holding time at temperature (s).

(18) $$h=4\cdot \sqrt{\left[3\times 10^{-7}\cdot exp(-20,904\cdot \frac{1}{T})\right]\cdot t}$$

For AISI 304 steel, the diffusion zone width can generally be determined for temperatures (*T*) in the range of 1223.15 to 1423.15 K and for times (*t*) in the range of 1 to 5 h, according to Equation (19).

(19) $$h=4\cdot \sqrt{\left[5\times 10^{-7}\cdot exp(-21,254\cdot \frac{1}{T})\right]\cdot t}$$

For the limit cases (950 °C_1 h and 1150 °C_5 h), using the above-described generalized equations, the maximum deviations in the calculation are as follows: For AISI 304 steel, at 950 °C_1 h, it is 7.7 µm, and at 1150 °C_5 h, it is 14.6 µm. For AISI 316L steel, at 950 °C_1 h, it is 8.2 µm, and at 1150 °C_5 h, it is 11.2 µm. From the above, it is evident that for temperatures in the range 950 to 1150 °C and for times in the range 1 to 5 h, the generalized equations for calculating the diffusion zone between austenitic steel and Ni interlayer can be used with sufficient accuracy.

The knowledge of diffusion coefficients, and especially the information about the width of the diffusion zone, is very important when preparing joints using interlayers. This helps to optimize the diffusion joint both in terms of the process parameters used and in terms of reducing the heterogeneity of the joint. This not only affects the mechanical properties of the joint but also other secondary properties, especially physical properties. It also determines the value and type of residual stresses in the joint due to the different thermal expansions.

Nickel interlayers are generally most used in the diffusion bonding of austenitic stainless steel and titanium alloys [5], and therefore our next aim is to determine the diffusion coefficients into titanium alloys and also to determine the diffusion coefficients of vanadium and niobium into the above materials.

## 5. Conclusions

The study presented in this paper aimed to study the diffusion kinetics of nickel into austenitic stainless steels, AISI 304 and AISI 316L, and also to determine the diffusion coefficients and then the widths of the diffusion zones for the most commonly used temperature ranges and holding times. The results obtained can be summarized in the following points:

(1) Temperature has a significant effect on the diffusion kinetics and, therefore, the depth of diffusion of nickel into the austenitic steel. It is also dependent on the holding time and temperature.
(2) Nickel diffuses into both the austenitic steels, AISI 304 and AISI 316L, at approximately the same rate, although AISI 316L has approximately 5% more Ni and 2.2% more Mo.
(3) Diffusion already occurs during the heating and subsequent cooling of the sample. In the case of a temperature of 950 °C for austenitic steels, it is between 5.6 and 7.3% of the total diffusion depth. At 1050 °C, it is 6.8–12.6%, and at 1150 °C, it is 11.7–23.8% of the total diffusion depths measured at holding times of 1 and 5 h.
(4) To determine the diffusion coefficient of Ni into specific steels, generalized Equations (16) and (17) were formulated for a temperature range of 1223.15–1423.15 K, which can be used with sufficient accuracy to determine the widths of the diffusion zones at the interface between austenitic steel (AISI 316L/304) and the Ni interlayer.
(5) The calculated diffusion depths were different from the real values in the range of 5% to 28.6%. The largest differences were at 950 °C. Although the deviation from the real value seems to be quite high, the real difference in diffusion zone width at 950 °C is a maximum of 6.7 µm.
(6) The diffusion zone width, and therefore the basic information for optimizing the interlayer thickness, can be determined with sufficient accuracy for AISI 304 steel generally for temperatures (T) in the range 1223.15–1423.15 K and for times (t) in the range 1 to 5 h according to Equation (18) and for AISI 316L steel according to Equation (19).

## Figures and Tables

**Table 1:** Chemical composition (wt%) of the AISI 304 steel.

AISI 304		C	Cr	Mn	Ni	Si	S	P	N
EN 10088-1	min.	-	17.50	-	8.00	-	-	-	-
	max.	<0.07	19.50	2.00	10.50	1.00	0.015	0.045	<0.11
Experiment		0.045	18.37	1.66	8.11	0.23	0.013		0.077

**Table 2:** Chemical composition (wt%) of the AISI 316L steel.

AISI 316L		C	Cr	Mn	Mo	Ni	Si	S	P	N
EN 10088-1	min.	-	16.50	-	2.00	10.00	-	-	-	-
	max.	<0.03	18.50	2.00	2.50	13.00	1.00	0.015	0.045	<0.11
Experiment		0.03	18.50	1.96	2.24	13.10	1.02	0.02		0.074

**Table 3:** Tensile test results for basic materials—AISI 304 and AISI 316L.

Sample No.	R_p0.2_ [MPa]	UTS [MPa]	A_g_ [%]	A_40_ [%]
AISI 304	445.5 ± 7.3	643.0 ± 3.2	33.60 ± 0.16	42.03 ± 0.09
AISI 316L	512.7 ± 5.2	662.5 ± 7.1	24.17 ± 0.61	39.47 ± 0.11

**Table 4:** Overview of temperature treatments.

Sample No.	Temperature [°C]	Time [Hours]
1	950	1
2	950	5
3	1050	1
4	1050	5
5	1150	1
6	1150	5

**Table 5:** Calculated diffusion coefficients.

D_Ni_ [m^2^·s^−1^]	1 h		5 h	
	304	316L	304	316L
950 °C	3.90 × 10^−15^	3.89 × 10^−15^	1.26 × 10^−15^	9.69 × 10^−16^
1050 °C	8.28 × 10^−15^	9.15 × 10^−15^	5.47 × 10^−15^	5.18 × 10^−15^
1150 °C	2.88 × 10^−14^	2.81 × 10^−14^	2.30 × 10^−14^	1.64 × 10^−14^

**Table 6:** Results of two-factor analysis of variance ANOVA with repetition.

Factor	AISI 304		AISI 316L	
	*p*-Value	Evaluation	*p*-Value	Evaluation
Time	0.0014	Has an effect	1.62 × 10^−5^	Has an effect
Temperature	4.92 × 10^−16^	Has an effect	8.49 × 10^−15^	Has an effect

**Table 7:** Diffusion depths from real samples.

h [µm]	0 h		1 h		5 h	
	304	316L	304	316L	304	316L
950 °C	1.46	1.31	19.89	20.04	25.08	23.41
1050 °C	3.15	3.04	25.08	31.68	41.80	45.02
1150 °C	10.72	9.41	45.09	44.96	71.90	80.26

**Table 8:** Calculated diffusion depths according to Equation (14).

h [µm]	1 h		5 h	
	304	316L	304	316L
950 °C	14.99	14.97	19.03	16.71
1050 °C	21.83	22.96	39.68	38.61
1150 °C	40.73	40.24	81.30	68.73

**Table 9:** Comparison of real and calculated diffusion depths.

∆h [µm]	1 h		5 h	
	304	316L	304	316L
950 °C	4.90	5.07	6.05	6.70
1050 °C	3.25	8.72	2.12	6.41
1150 °C	4.36	4.71	9.40	11.54

## Data Availability

Not applicable.
